# Epidemiology and Prevalence of Oral Candidiasis in HIV Patients From Chad in the Post-HAART Era

**DOI:** 10.3389/fmicb.2022.844069

**Published:** 2022-02-17

**Authors:** Liliane Taverne-Ghadwal, Martin Kuhns, Timo Buhl, Marco H. Schulze, Weina Joseph Mbaitolum, Lydia Kersch, Michael Weig, Oliver Bader, Uwe Groß

**Affiliations:** ^1^Institute for Medical Microbiology and Virology, University Medical Center Göttingen, Göttingen, Germany; ^2^Clinic for Dermatology, University Medical Center Göttingen, Göttingen, Germany; ^3^Association Interdiocésaine de Lutte contre le Sida (AILS), N'Djamèna, Chad; ^4^Medical Health Center of Maingara, Belacd de Sarh, Chad

**Keywords:** oral *Candida* colonization, HIV, AIDS, Chad, NNRIT-HAART

## Abstract

Oral candidiasis remains a common problem in HIV-infected individuals, especially in sub-Saharan Africa. Here, we performed the first study in Chad on the prevalence of oral yeasts carriage and oral candidiasis in HIV-positive subjects from southern Chad and analyzed the influence of HAART, CD4^+^ T-cell numbers, and antimycotics in 589 patients. These patients were recruited from a specialized medical center for HIV patients in Sarh and from a rural medical health dispensary in the vicinity, including a total of 384 HIV-positive and 205 HIV-negative individuals. Yeasts obtained from oral specimen were identified by MALDI-TOF MS and their antifungal susceptibility profiles determined. The overall prevalence of yeast colonization and symptomatic oral candidiasis in HIV-infected patients was 25.1%. The prevalence of oral candidiasis was higher in untreated than in HAART-treated HIV-positive patients (16% vs. 2%; *p* < 0.01). Oral candidiasis was furthermore associated with high fungal burdens of *Candida albicans* and a CD4^+^ T-cell number <200/μl. A shift toward non*-albicans Candida* species was observed under nucleoside-based HAART therapy. Azole antifungal drug resistance was only observed for the intrinsically resistant species *Candida krusei* and *Candida glabrata*. Prevalence of oral candidiasis in the studied area was very low. The species distribution was similar to other countries around the world, with *C. albicans* being dominant. *Candida dubliniensis* was not isolated. Nucleoside-based HAART therapy significantly reduced oral colonization as well as occurrence of oral candidiasis caused by *C. albicans* and led to a species shift toward non-*albicans* species. Antifungal resistance was not yet a concern in Chad.

## Introduction

Oral candidiasis is one of the most common oral lesions associated with human immunodeficiency virus infection (Holmberg and Meyer, 1986; Phelan et al., 1987; Barr, 1992; Laskaris et al., 1992; Greenspan et al., 2000; Leao et al., 2009; Chopra and Arora, 2012). It can be the first hint to the manifestation of AIDS (Klein et al., 1984; Laskaris et al., 1992; Chapple and Hamburger, 2000) and is strongly associated with esophageal candidiasis (Tavitian et al., 1986), one of the AIDS-defining illnesses (Dore and Cooper, 2001). *Candida albicans* is a commensal of the human gastrointestinal tract and oral mucosa. It is the most common yeast causing oropharyngeal candidiasis (Schoofs et al., 1998), but other non-*albicans Candida* species have also emerged in this context (De Bernardis et al., 1996; Powderly et al., 1998; Cartledge et al., 1999; Mushi et al., 2016, 2018; Ambe et al., 2020). Colonization of oral mucosal surfaces with yeasts such as *C. albicans* is closely correlated to symptomatic disease (oropharyngeal and esophageal candidiasis; Pappas et al., 2003) and latter one with the severity of cellular immunodeficiency, especially infected hosts with the HI virus (Mercante et al., 2006; Malele Kolisa et al., 2019). In a resource-poor setting without access to CD4^+^ T-cell counting and HIV viral load measurements, oral candidiasis is one of the most important clinical markers of HIV infection, disease progression, CD4^+^ T-cell status (Fidel, 2006; Berberi et al., 2015), and can even give a hint to antiretroviral therapy failure (Hodgson and Rachanis, 2002; Ramirez-Amador et al., 2007).

If left untreated, these lesions contribute considerably to HIV-associated morbidity (Holmberg and Meyer, 1986; Gautam et al., 2010). The prevention and treatment of oral candidiasis is therefore an important component of the maintenance of the quality of life of affected individuals (Minamoto and Rosenberg, 1997).

Between 67% and 70% of individuals infected with HIV worldwide live in Sub-Saharan Africa (Hodgson and Rachanis, 2002; UNAIDS, 2009), but there are only few reports on oral candidiasis or antifungal drug resistance from this region. Reports from Tanzania, Mali, Ghana, Uganda, Cameroon, Ivory Coast, and South Africa show that oral candidiasis still is significantly associated with HIV infection (Hamza et al., 2008; Agwu et al., 2012; Tami-Maury et al., 2012; Kwamin et al., 2013; Konate et al., 2017; Malele Kolisa et al., 2019; Ambe et al., 2020).

In the general population of Chad, the HIV prevalence amounts to approximately 3.4% (UNAIDS, 2009), but may be as high as 10% in urban areas, such as Sarh. As in other Sub-Saharan countries (Hodgson and Rachanis, 2002), sampling of the oral cavity for determination of fungi, and to an even lesser degree antifungal drug resistance testing, is not conducted on a regular basis. Diagnosis and treatment of this important opportunistic infectious disease are based on very limited knowledge and, for the most part, rely solely on clinical impression, which, however, is not always conclusive. So far, there are no data available on the prevalence of oral yeast colonization and infection, species distribution, or antifungal drug susceptibility among HIV patients of Chad.

Here, we conducted a cross-sectional study to determine the prevalence of oral yeast colonization and infection among HIV-infected and HIV-negative subjects in Chad. Furthermore, we evaluated the susceptibility of the identified isolates to antifungal drugs and analyzed the association of oral candidiasis with the degree of immunosuppression and the effects of nucleoside-based HAART and antimycotics on the oral fungal burden.

## Materials, Methods, and Patients

### Patient Recruitment

This study was approved by the participating institutions in Chad and the ethical committee of the University Medical Center Göttingen, Germany (21/06/07). All patients involved were informed about the aim of the study and gave their informed consent according to the Helsinki Declaration before inclusion into the study (World Medical Association, 2013).

Patients were recruited from individuals presenting for consultation to the clinic of Maingara in Sarh, the third largest city of Chad. The majority of these patients were either HIV-infected and came to their regular monthly health controls or had just been tested HIV-positive. Patients from a dispensary in Bemouli, a small health center in a rural area 50 km away from Sarh, were included into the study to enlarge the HIV-negative control group. HIV status in both groups was obtained by testing with the HIV test kit Determine HIV-1/2 (Abbott Diagnostic Medical Co. Ltd., Matsudo, Japan), and if positive, confirmed through the rapid test kit ImmunoComb II HIV 1&2 Bispot (Orgenics, Yavne, Israel). HIV positivity was only considered when both tests were positive. Both study areas are located in the subtropical South of Chad.

### Anamnesis and Sampling

Sampling was performed over a period of 3 months. It included a short anamnesis, taking data on age, sex, HIV status, current, and previous opportunistic infections and medications, antiretroviral therapy, and the latest CD4^+^ T-cell count were directly recorded or taken from medical records. Additionally, a brief clinical examination was done, including inspection of the oral cavity. From patients presenting again during the study period, a consecutive sample and examination were taken to evaluate disease progression and effect of antimycotics or HAART therapy. Only samples from the first visit of each patient were considered for evaluation of the prevalence of fungal colonization and patients who had received antifungal or antibiotic treatment within 3 weeks before examination were excluded from this analysis. Similarly, patients having received less than 4 weeks of HAART therapy were excluded from the HAART^+^ group. The oral cavity of the patients was sampled by taking swabs with a sterile cotton swab (Copan, Brescia, Italy) from visible oral lesions, or when no symptoms were visible, going over tongue, hard gum, and side cheek pockets. The swab was then directly inoculated onto Sabouraud agar (Oxoid, Wesel, Germany). Due to the lack of an incubator, the plates were cultured at room temperature (26°C–28°C during nighttime, 30°C–36°C during daytime) and controlled for the growth of yeasts after 24 and 48 h. Yeast growth was confirmed by identification under the microscope. For evaluation of the fungal burden, colony-forming units (CFU) were counted (Pomarico et al., 2009). CFU counts were categorized into seven semi-quantitative classes (0: no growth; 1: single colony, 2: 2–5 CFU, 3: 6–10 CFU, 4: 11–15 CFU, 5: 16–25 CFU, 6: >25 CFU, or 7: at least partial confluent growth on a 1/8th of an agar plate). Categories 1–4 were considered as “low fungal burden” and categories 5–7 as “high fungal burden.” Samples of several colonies were generously taken from the plates including colonies of morphologically distinct appearance and stored on Sabouraud agar slants at 4°C until they were transferred to Germany.

### Diagnostic Criteria

**Figure 1:**
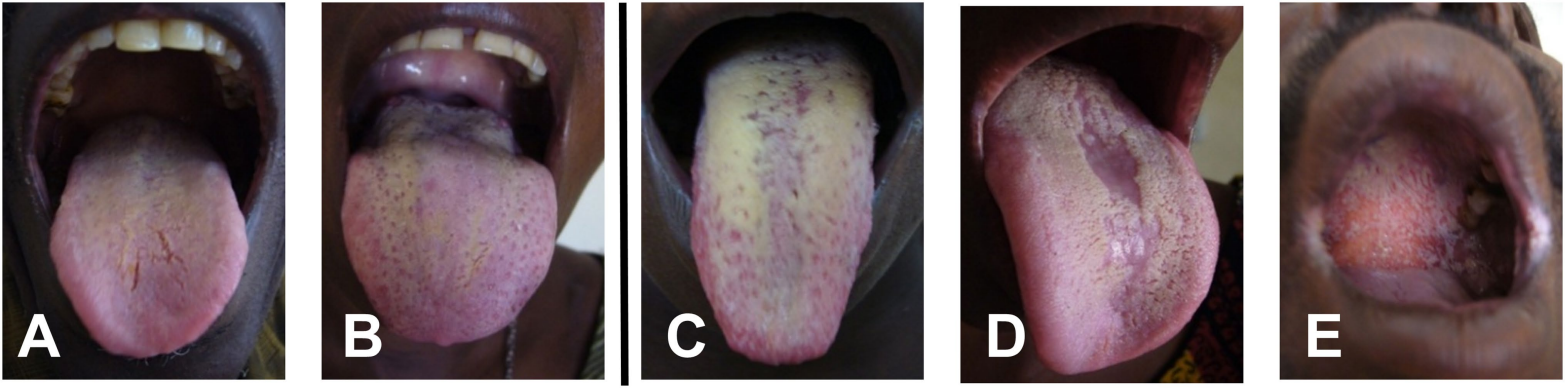
Representative samples of oral symptoms of the tongue and palate within the study group: **(A,B)** mild symptoms with less than 50% coverage of the tongue by whitish plaque, **(C)** >50% of tongue surface covered with whitish plaque, **(D)** median rhomboid glossitis with central atrophy, and **(E)** involvement of palate.

Based on the diagnostic criteria proposed by Parihar (2011) and the scoring index for oral mucositis proposed by McGuire et al. (2002), oral candidiasis was classified based on clinical and mycological observations. Confirmatory tests such as exfoliative cytology or tissue biopsy were not available on site. Patients were divided into four distinct subgroups: (a) asymptomatic, (b) mildly symptomatic, for example, a whitish or yellowish coated tongue with <50% affected area but no further clinical signs, (c) moderately symptomatic, for example, a whitish or yellowish coated tongue with >50% affected area without erythematous ground, and (d) severely symptomatic, for example, thrush and/or atrophy and/or erythema and/or other mucosal sites affected like palate or side cheek pockets (Figure 1).

Together with the results from mycological culture (colonized vs. non-colonized and CFU counts), four different patient groups were defined: (i) yeast free irrespectively of clinical symptoms (non-carriers), (ii) culture-positive but asymptomatic (asymptomatic yeast carriers), (iii) culture-positive with mild symptoms (mildly) or moderate symptoms and low fungal burden, and (iv) culture-positive with moderate or severe symptoms with high fungal burden. Only patients of group (iv) were classified as having oral candidiasis. Yeast-positive patients with mild coating as well as yeast-positive patients with moderate coating but low fungal burden were considered colonized.

### Yeast Differentiation and Drug Susceptibility Testing

In Germany, samples were recultivated on Sabouraud agar (Oxoid, Wesel, Germany) and those presenting with apparent mixed cultures by colony morphology separated and recultivated until obtaining morphological purified cultures. Isolated purified species where again identified as yeast under the microscope, then further processed with phenotypic methods (rice and Staib agar, API) and PCR in difficult cases. Additionally, all samples (live isolates) were typed and confirmed by MALDI-TOF MS (Bruker MALDI Biotyper 3.0) as described previously (Bader et al., 2011) and stored using the Cryobank system (Mast Diagnostica, Reinfeld, Germany) at −70°C. Fifteen isolates could not be re-cultured and were omitted from the resistance analyses. In nine of these, the species could be determined by sequencing of the ITS2 locus (Chen et al., 2000) amplified from DNA prepared from the storage swab.

Antifungal susceptibility testing was determined by the CLSI broth microdilution method M27-A3 (CLSI, 2008). Antimycotics tested were fluconazole, itraconazole (Discovery Fine Chemicals, Bournemouth, United Kingdom), nystatin, amphotericin B (Sigma, Taufkirchen, Germany), and caspofungin (MSD, Whitehouse St., NJ, United States). The minimal inhibitory concentration (MIC) for amphotericin B and nystatin was defined as the lowest concentration in which at least 90% of growth of the sample was inhibited, defined as MIC_90_, for caspofungin and the azoles, as the lowest concentration in which at least 50% of growth was inhibited (MIC_50_). For the quality control of the plates, the strains recommended by CLSI [*C. parapsilosis* (ATCC 22019) and *C. krusei* (ATCC 6258)] were included in the testing procedure. Species-specific breakpoints were used in accordance with the CLSI guidelines (CLSI, 2017). For polyenes such as nystatin no clinical breakpoints have yet been defined, and use of itraconazole breakpoints is discouraged. Briefly, *C. albicans*, *C. krusei*, and *C. tropicalis* were considered caspofungin susceptible at MIC values ≤0.25 mg/L, *C. glabrata* at ≤0.125 mg/L, and *C. parapsilosis* group isolates at ≤2 mg/L. For fluconazole, *C. albicans*, *C. parapsilosis* group, and *C. tropicalis* were considered susceptible at MIC values ≤2 mg/L, and *C. glabrata* at ≤32 mg/L. *C. krusei* is considered intrinsically resistant to fluconazole.

### HIV Testing, CD4^+^ T-Cell Counting, and Antiretroviral Therapy

The HIV status and the latest CD4^+^ T-cell counts of the patients were taken from hospital records. HIV testing was done as described above. CD4^+^ T-cell counts were routinely determined (cyFlow, Partec, Münster, Germany) during regular monthly health check-ups. Only recent CD4^+^ T-cell counts from the 3 months prior to the first consultation and during the study period were considered for evaluation, therefore, although patients were encouraged to regularly consult the clinic, a current CD4^+^ T-cell count was not available for all.

Patients received antiretroviral treatment (HAART) according to the national guidelines of Chad for antiretroviral therapy, which refer to the WHO Standard (WHO, 2006). Briefly, HAART was indicated when the patient had a CD4^+^ T-cell count <200 cells/μl or was in WHO clinical stages IV or III with CD4^+^ T cells <350 cells/μl. Treatment could also be considered in patients in WHO clinical stage II when CD4^+^ T-cell counts were <350 cells/μl. The antiretroviral therapy available in Maingara at time of this study was TRIOMUNE, a combination of stavudine, lamivudine, and nevirapine, given twice daily. In cases of intolerability of nevirapine or tuberculosis treatment with rifampicin, patients received a combination with either efavirenz (four cases) or indinavir (four cases) instead of nevirapine.

Only samples from the first visit of each patient were considered for evaluation of the prevalence of fungal colonization and patients who had received antifungal or antibiotic treatment within 3 weeks before examination were excluded from this analysis. Similarly, patients having received less than 4 weeks of HAART therapy were excluded from the HAART+ group.

### Statistics

Statistical significance was calculated using chi-square and Student’s *t*-test, where value of *p* < 0.05 were considered as significant.

## Results

### Patients

**Figure 2:**
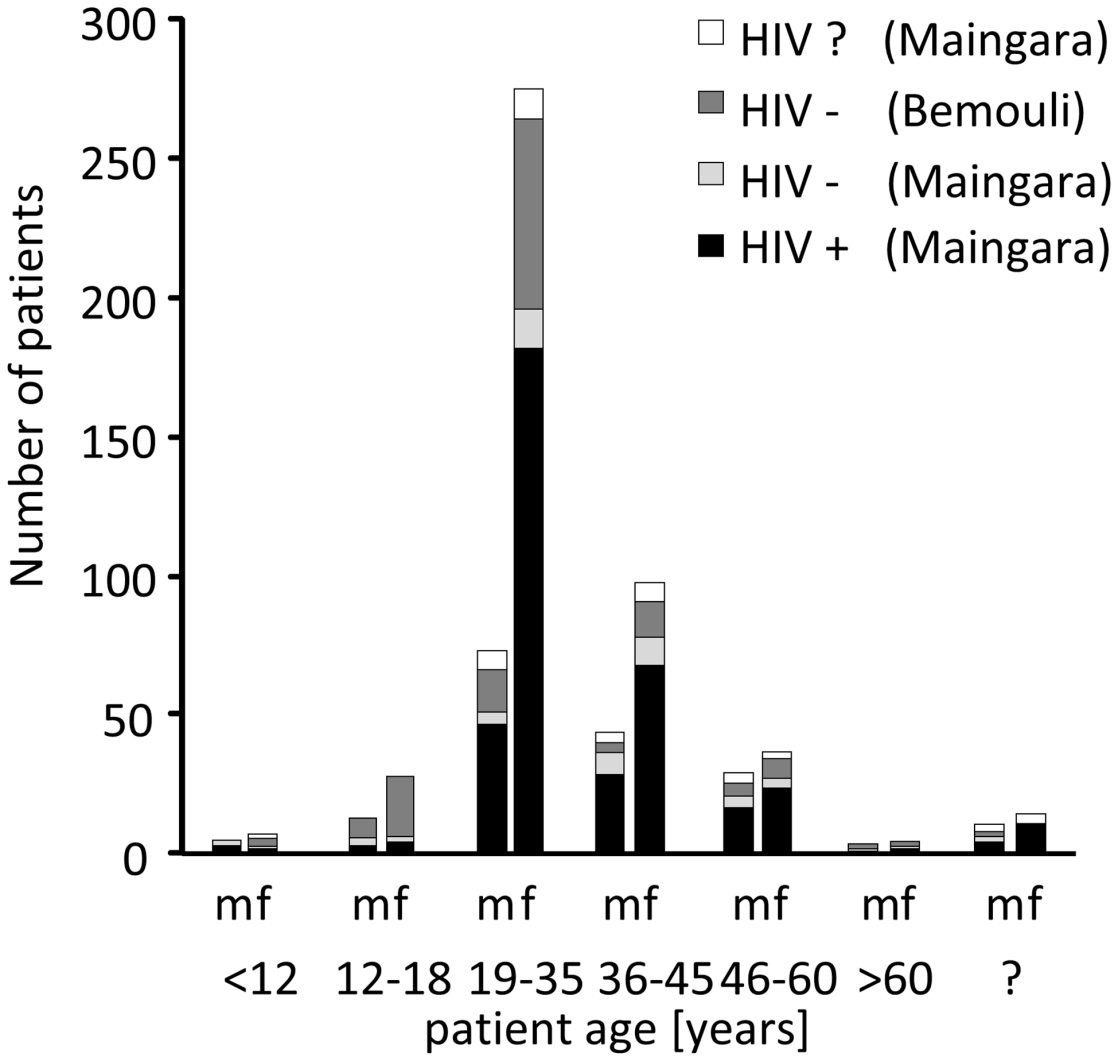
Distribution of the patients according to age, gender, and HIV status. m, male; f, female; and ?, unknown.

During the study period 589 patients were seen; 441 at the clinic in Maingara (Sarh) and 148 at the medical dispensary of Bemouli (Figure 2). In Maingara 87.1% of the patients were HIV-infected (*n* = 384), 27.8% HIV-negative (*n* = 57). As there were no relevant significant differences between the HIV-negative patients from Maingara and Bemouli (data not shown), these patients were combined into one group. The mean average age was 34 in Maingara and 28 in Bemouli (Figure 2). In all subgroups, women were overrepresented (>70%). This bias is similar to reports from other African countries (Hamza et al., 2008; Agwu et al., 2012; Tami-Maury et al., 2012; Kwamin et al., 2013; Konate et al., 2017; Ambe et al., 2020), since women are more often affected by HIV than men and also more likely to consult the local health care system (UNGASS, 2008; UNAIDS, 2009). Since there were no specific or relevant differences observed between male and female subgroups, genders were not further separated for our analyses.

### Prevalence of Clinical Manifestations of Oral Candidiasis and Colonizing Yeasts

The majority of patients with clinical signs of oral candidiasis (Table 1; Figure 1) presented with a whitish coated tongue affecting >50% of the area without further signs of inflamed erythematous area beneath the coating (Figure 1). The second most common clinical sign was the median rhomboid glossitis (MRG), out of which the majority appeared in the HAART− group with a prevalence of 26.6% and a mean CD4^+^ T-cell count of <150 cells/μl. There were five cases of acute pseudomembranous candidiasis (PC; whitish creamy lesions on erythematous surface) with additionally affected palate. Two patients without HAART presented with acute PC combined with either MRG or atrophy.

**Table 1:** Distribution of symptoms according to semi-quantitative yeast culture results.

	Patient classification		HIV−			HIV+/HAART−			HIV+/HAART+		
	Fungal burden		Neg	Low	High	Neg	Low	High	Neg	Low	High
Symptoms	None	-	89	16	5	41	4	2	56	6	1
	Mild	Coated tongue <50%	22	3	5	24	5		35	8	1
	Moderate	Coated tongue >50%	19	2	3	13	7	4	12	5	
	Severe	Acute PC			1			5^a^			2
		MRG		2		3	2	7^a^	1	1	
		Atrophy	1			1		3^a^			

a *Includes two patients with acute PC combined with either MRG or atrophy*.

**Figure 3:**
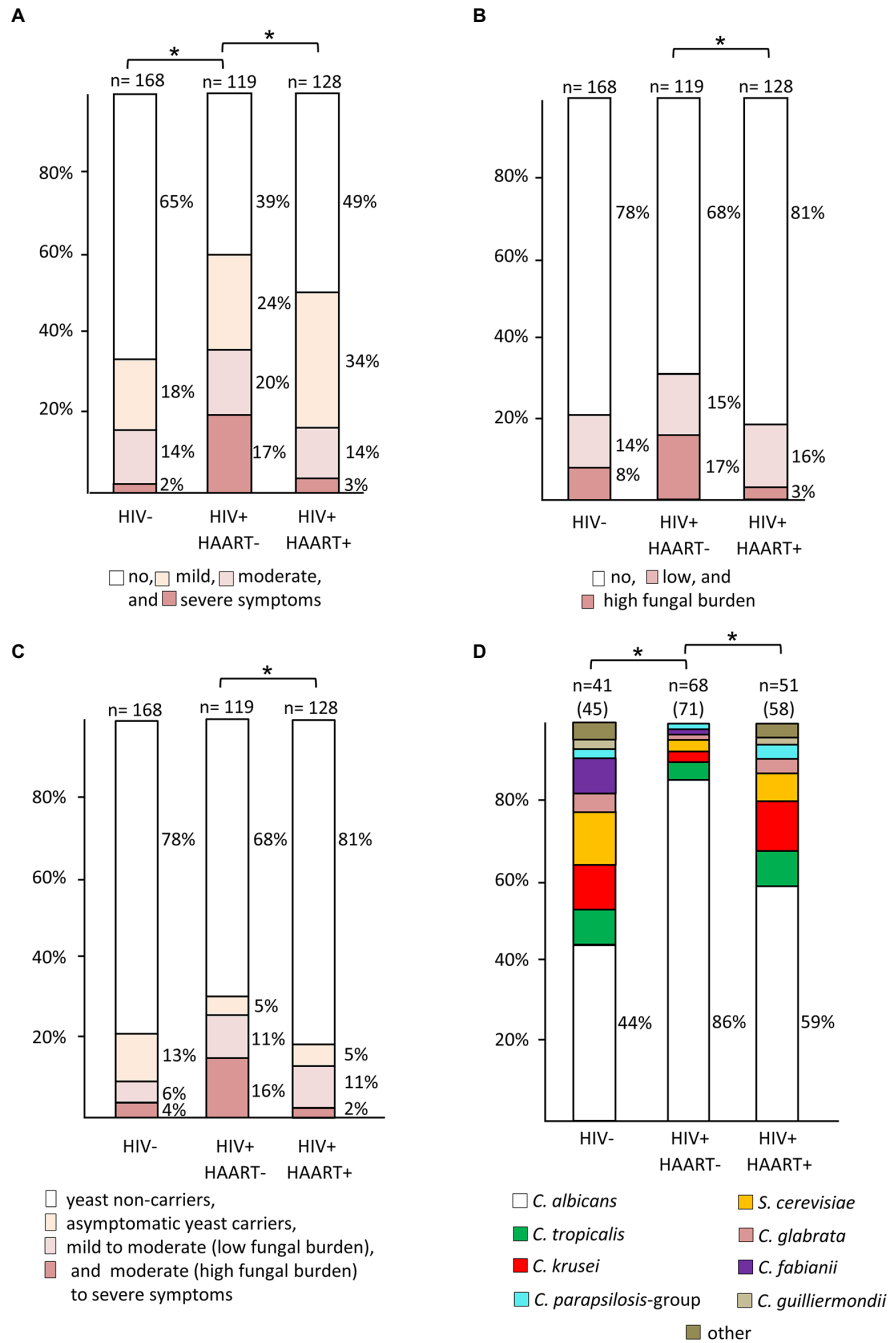
Prevalence rates of yeast species among patient groups with different HIV and treatment status. **(A)** Rates and degree of oral symptoms (* indicates significant differences in prevalence of moderate-to-severe symptoms), **(B)** rates and degree of oral yeast colonization (* indicates significant differences in prevalence of a high yeast burden), **(C)** association of oral colonization with disease symptoms (* indicates significant differences in moderately to severely symptomatic yeast carriers), and **(D)** species distribution derived from yeast-positive swabs. In panels **(A**–**C)**, numbers above columns indicate size of patient subgroups. a: Y-axis of panel **(D)** is scaled to total number of isolates, given in parentheses after number of swabs. Due to mixed-species colonization the number of isolates can exceed number of swabs (* indicates significant differences in prevalence rates of *Candida albicans*).

The prevalence of moderate-to-severe symptoms (Figure 3A) was significantly higher in the HIV+/HAART− group than in the HIV+/HAART+ (*p* < 0.01) and the HIV-negative group (*p* = 0.01). HIV+/HAART+ had again similar rates of severe symptoms as the HIV− control group.

The overall prevalence of a high yeast burden (as measured by semi-quantitative CFU counts) in the oral cavity from HIV+/HAART− was significantly higher than among those with HAART therapy (*p* < 0.01) but not as compared to the HIV-negative control group (*p* = 0.09; Figure 3B). HIV+/HAART+ patients had similar rates of oral yeast colonization as HIV-negative patients, with this group also having the lowest oral fungal burden (19% vs. 22% (HIV−) and 32% (HIV+/HAART−); Figure 3B).

No or mild symptoms were highly associated with no or low yeast growth (Table 1) in HIV-positive patients (*p* < 0.01). In contrast, moderate-to-severe symptoms were not necessarily associated with the isolation of yeasts in HIV+/HAART+ patients. Only 38% of patients with moderate-to-severe symptoms in the HIV+/HAART+ compared to 62% in the HIV+/HAART− group were yeast-positive.

HIV-positive patients with moderate-to-severe symptoms and a positive swab culture with high CFU were classified as having oral candidiasis. Hence, the prevalence rates were 16% in the HIV+/HAART− and 2% in the HIV+/HAART+ group (*p* < 0.01; Figure 3C).

*Candida albicans* was the most frequently isolated species in our study, being highly dominant in the HIV+/HAART− group (86%) as compared to the HIV-negative (44%; *p* < 0.01), and HIV+/HAART+ group (59%; *p* < 0.01; Figure 3D). Distributed across all patient subgroups the next most prevalent species isolated were *C. krusei* (*Issatchenkia orientalis*), followed by *C. tropicalis*, and, surprisingly, the non-*Candida* yeast species *Saccharomyces cerevisiae*. The frequency of *C. glabrata* was very low, and it was not found in the HIV-negative group from Bemouli at all. However, *C. fabianii* (*Pichia fabianii*) and the non-*Candida* yeast species *S. cerevisiae* were the second next most prevalent species there (Figure 3D), two yeast species only rarely giving rise to clinical symptoms. *C. dubliniensis*, which has been linked to OC in HIV patients (Gutiérrez et al., 2002), was not isolated from any of the patients, irrespectively of HIV status.

In some cases, mixed cultures of two to three species were observed: four cases in the HIV-negative group including the species *C. albicans*, *C. glabrata*, *C. tropicalis*, *C. krusei*, and *S. cerevisiae*, seven in the HIV+/HAART+ and three in the HIV+/HAART− group. In the HIV+/HAART+ group mixed colonization included predominantly a combination of non-*albicans* species (*C. tropicalis*, *C. krusei*, *C. parapsilosis*, *C. orthopsilosis*-like, *C. kefyr*, *C. glabrata*) whereas in the HIV+/HAART− group mixed colonization always included *C. albicans* combined either with *C. tropicalis*, *C. krusei* or *C. parapsilosis*.

### Influence of HAART and CD4^+^ T-Cell Numbers on Oral Yeasts

Among the HIV-positive patients where CD4^+^ T-cell counts were available (*n* = 252), we analyzed the association of HAART therapy and CD4^+^ T-cell counts with oral symptoms and the degree of oral yeast colonization. Any patients receiving antifungal treatment were excluded from this analysis.

Irrespectively of the degree of symptoms high fungal burden was significantly associated with CD4^+^ T-cell counts ≤200 cells/μl in HIV+/HAART+ and HIV+/HAART− patients (*p* < 0.01).

**Figure 4:**
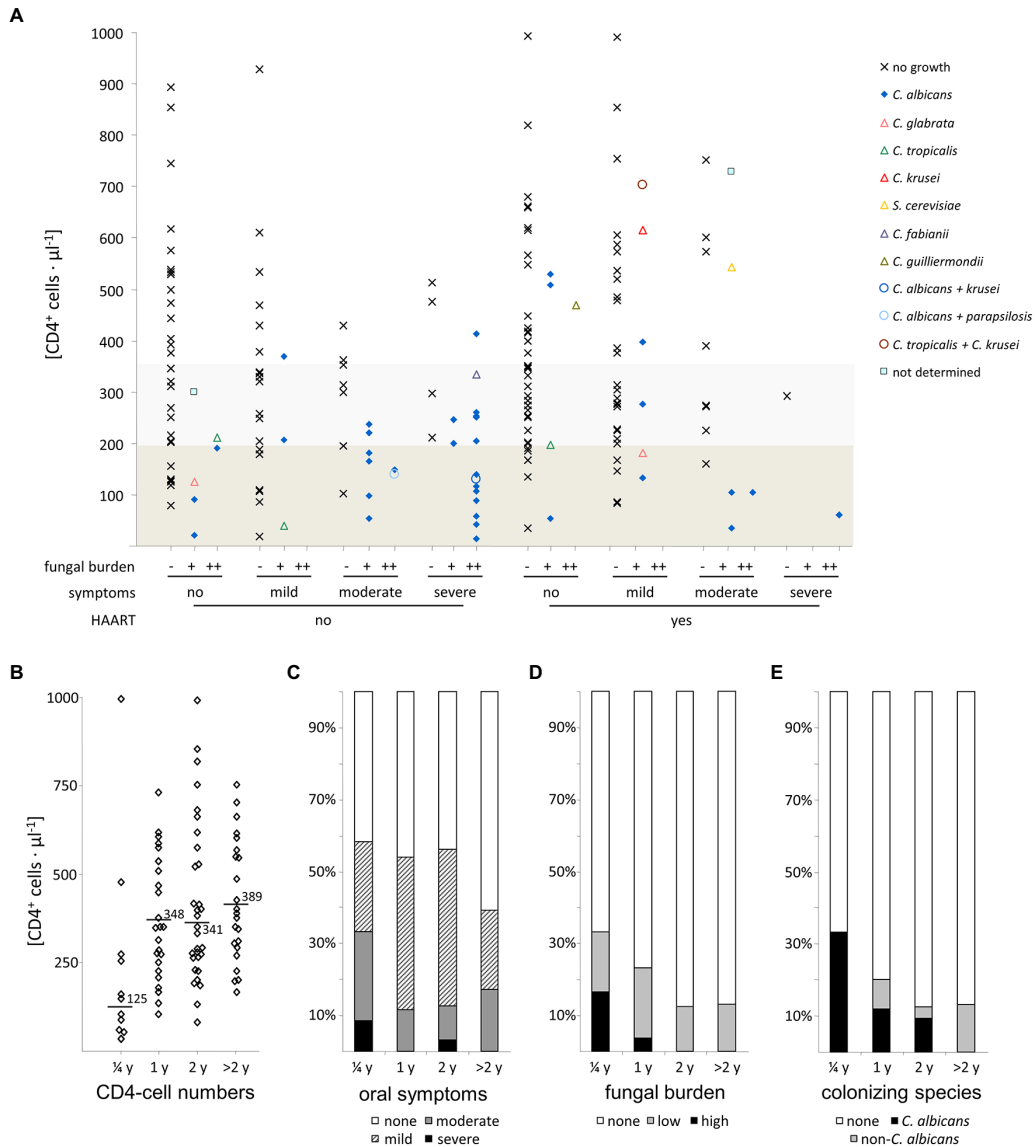
Influence of Triomune HAART therapy on oral symptoms and yeast colonization in HIV-positive patients. **(A)** Association of CD4^+^ T-cell numbers with observational parameters investigated, **(B)** CD4^+^ T-cell numbers, **(C)** oral symptoms, **(D)** fungal burden, and **(E)** colonizing yeast species observed in HIV-positive patients with different lengths of Triomune HAART therapy (up to 0.25, 1, 2, and more than 2 years after initiation). Boxed regions in panel **(A)** denote CD4^+^ T-cell numbers below 200 cells/μl (dark grey) and 200–350 cells/μl (light grey). Numbers in panel **(B)** are mean values.

In both HAART+ and HAART− patients with oral candidiasis we found *C. albicans*, alone or in combination with non-*albicans* species. In patients not receiving antiretroviral therapy, mixed colonization always appeared in a combination with *C. albicans* and with CD4^+^ T-cell counts <200 cells/μl (Figure 4A). Interestingly, colonization with *C. glabrata* or *C. tropicalis* was seen only in patients with a CD4^+^ T-cell count <200 cells/μl in both groups but without severe clinical symptoms. Other species such as *C. guilliermondii*, *C. krusei*, and *C. parapsilosis* group species were mainly found at low abundance when CD4^+^ T-cell counts were >300 cells/μl. The same was true for the non-*Candida* yeast species *S. cerevisiae* that was probably a transient organism in isolated cases.

For those patients where no growth of yeasts was found in the oral cavity, there was no significant difference in CD4^+^ T-cell counts between HIV-infected patients under HAART and without antiretroviral therapy (*p* > 0.5).

The majority of the HIV-infected patients receiving antiretroviral therapy without presence of yeasts in the oral cavity had been receiving HAART already for over 1 year. Division of the patients into subgroups according to the length of HAART therapy (Figures 4B–E) showed that groups with at least 1 year of treatment had higher CD4 counts than the group with 3 months of treatment, as well as a lower frequency of severe oral symptoms, colonization, and high fungal burden, as well as reduced colonization specifically with *C. albicans* and a rise in colonization with non-*albicans* species (Figure 4E). The presence of oral candidiasis is not completely eradicated after HAART for 1 year, cases are still seen even with a treatment exceeding this time (Figures 4A–D). Similarly, colonization with yeasts in symptom-free patients and cases with high colonization are still present.

### Antifungal Treatment and Drug Susceptibility

**Figure 5:**
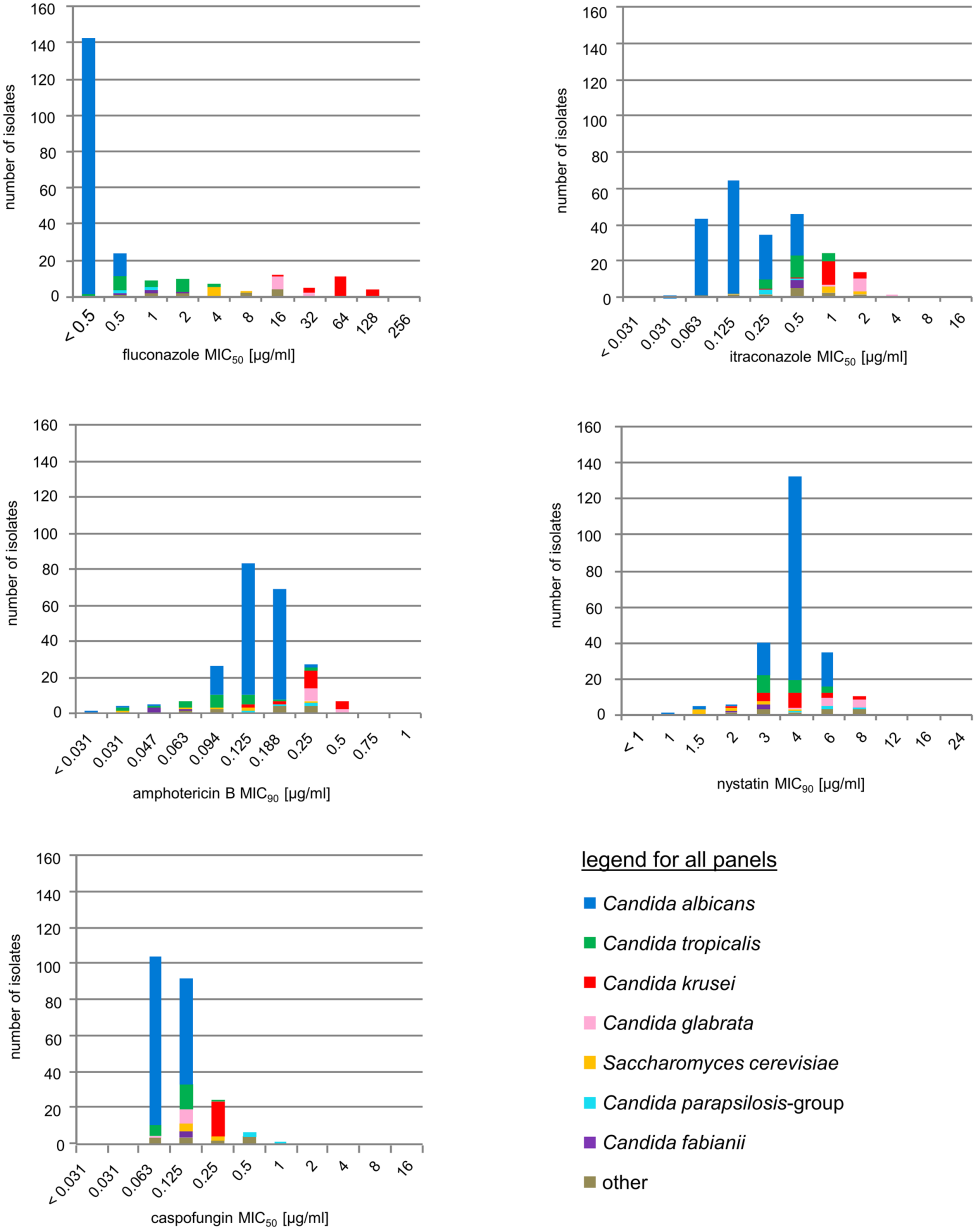
Antifungal drug resistance. Susceptibility distribution to five common antifungals, stratified by species. For color codes see inset, species-specific breakpoints are briefly outlined in the Methods section.

Antifungal susceptibility testing showed that no particularly unusual resistance phenotype was present among the isolates obtained in this study (Figure 5). If observed, higher MIC values were within the previously reported ranges for that particular species (Pfaller et al., 2006, 2010, 2011). Mainly, high MICs for azoles and polyenes were found in *C. krusei* and *C. glabrata*, however, the majority of patients harboring *C. glabrata* or *C. krusei* had no previous history of antifungal treatment (data not shown). Increased echinocandin MICs were only found for isolates from the *C. parapsilosis* group (*C. ortho*-, *meta*-, and *parapsilosis*), which, however, were within normal ranges observed for these species (Diekema et al., 2009). All colonized or infected patients without HAART but receiving antifungal therapy (*n* = 11) responded positively with reduction of oral colonization or symptoms (data not shown).

## Discussion

Oral candidiasis is a common opportunistic infection and often a first AIDS-defining disease in HIV-infected patients (Klein et al., 1984; Laskaris et al., 1992; Chapple and Hamburger, 2000). However, only a few studies on the prevalence of yeasts in the oral cavity of HIV-positive patients (Hodgson and Rachanis, 2002; Owotade et al., 2013; Africa and Abrantes, 2017) have been performed in sub-Saharan African countries. For Chad, no data are available at all. Here, we have studied the prevalence and epidemiology of oral asymptomatic and symptomatic yeast carriage of HIV-infected patients vs. non-HIV-infected individuals as controls from southern Chad and analyzed the impact of HAART and CD4^+^ T-cell numbers on oral yeast colonization and infection in HIV-infected individuals.

Historically, median rhomboid glossitis (MRG) was thought to be a developmental anomaly. However, in the 1970s *C. albicans was identified* in all patients presenting with median rhomboid glossitis giving evidence that MRG is caused by chronic fungal infection (Cooke, 1975; Wright, 1978). Starting in 1990, MRG was found to be common in HIV-infected patients, (Flaitz and Hicks, 1999; Barasch et al., 2000; Okunseri et al., 2003; Lalla et al., 2013). Very few studies highlight or distinguish between the appearance of the different candidiasis forms and study their epidemiology. A study performed in Zambia (Hodgson, 1997) described that erythematous candidiasis (EC) was associated with CD4^+^ T-cell counts <200 cells/μl. It seems that there is a correlation of the appearance of EC (and possibly MRG) with the absence of HAART treatment which is supported by our findings and other studies (Bodhade et al., 2011). In a study in Tanzania including patients under HAART the prevalence of EC was only 1.4% (Hamza et al., 2008).

Compared with other African studies, where the prevalence of oral candidiasis ranged from 42% in Cameroon (Ambe et al., 2020), 60% in Nigeria (Nweze and Ogbonnaya, 2011) 65% in Malawi (McCullough et al., 2001) up to 80% in South Africa (Patel et al., 2006), Ghana (Kwamin et al., 2013) and Ivory coast (Konate et al., 2017) the prevalence in the studied area in Chad was surprisingly low. These differences may be due to the differences in the selected study groups, diagnostic tools and criteria, and experience of the investigators. However, our data support previous findings (Jordan, 2007; Patel et al., 2012; Ambe et al., 2020) where investigations toward the correlation between oral manifestations and antiretroviral therapy found that oral *Candida* infections are less likely to develop when on HAART. This has been shown as well in a study among HIV patients in Nigeria, where the oral colonization rate and OC among HIV patients treated with HAART was only 20% and 0.5%, respectively, (Osaigbovo et al., 2017).

In our study, oral candidiasis emerged irrespectively of HAART when CD4^+^ T-cell counts were <200 cells/μl and was mainly caused by *C. albicans*. This is similar to reports from Tanzania (Matee et al., 2000), Ghana (Kwamin et al., 2013), Cameroon (Ambe et al., 2020), and India (Lattif et al., 2004; Anwar et al., 2012), where oral candidiasis is associated with CD4^+^ T-cell counts <200 cells/μl. Severe symptoms indicative of candidiasis were seen in only two patients with CD4^+^ T-cell counts >300 cells/μl. Whether the non-*albicans Candida* species present in both these cases were the cause of disease could not be clarified.

In the vast majority of cases based on the classification of having severe oral symptoms together with a yeast-positive oral swab lead us to suspect a CD4^+^ T-cell count <200 cells/μl. This monitoring strategy could help to initiate HAART and antifungal therapy in resource-poor settings. For patients who are not yet under HAART the probability that a patient presenting with severe oral symptoms is colonized by yeasts is high. In that group, 60% of the patients with severe symptoms were swab positive, had a mean CD4^+^ T-cell count <200 cells/μl, and were mainly infected with *C. albicans*.

In our study, the incidence of oral candidiasis significantly decreased under HAART like in other studies (Powderly et al., 1998; Yang et al., 2006; Lourenco et al., 2011; Ambe et al., 2020), but does not totally eliminate *Candida* from the oral microbiome, as has been described before (Cauda et al., 1999; Yang et al., 2006). With ongoing time of HAART, a shift toward non-*albicans* species was observed, which also correlated with a rise in CD4^+^ T-cell numbers. As oral candidiasis caused by *C. albicans* is still highly associated with a CD4^+^ T-cell count <200 cells/μl even in patients with a longer period of time of HAART, it is likely that the improvement of the immune function under HAART with increased CD4^+^ T-cell counts and decreased viral loads may be responsible for the decrease of oral candidiasis caused by *C. albicans* (Fethi et al., 2005; Sanchez-Vargas et al., 2005; Fidel, 2006; Yang et al., 2006; Ortega et al., 2009; Wu et al., 2012; Ribeiro et al., 2015; Maheshwari et al., 2016) and the emergence of non-*albicans* species colonizing the oral cavity of HIV-infected patients (Nweze and Ogbonnaya, 2011; Maheshwari et al., 2016).

In our setting, a basic selection of drugs was available to treat and prevent the most common AIDS-related opportunistic diseases, including a limited supply of antimycotics. Antifungal treatment was administered when patients presented with dermatomycoses or oral thrush, or in some cases in the absence of clinical symptoms when CD4^+^ T-cell numbers were <200 cells/μl. Patients received either azoles (oral fluconazole or ketoconazole) or polyenes (mouthwash with amphotericin B or nystatin).

Previous studies suggested that repeated exposure to azole antifungal agents might predispose for colonization and infection by non-*albicans* species, caused through the selection of less susceptible species like *C. glabrata* or *C. krusei*, especially in patients suffering from oropharyngeal candidiasis (Schoofs et al., 1998; Cartledge et al., 1999; Hope et al., 2002; Snydman, 2003; Melo et al., 2004; Enwuru et al., 2008; Hamza et al., 2008; Agwu et al., 2012; Maheshwari et al., 2016; Africa and Abrantes, 2017). The restricted and rare use of antimycotics, due to restricted availability in Chad, may explain the absence of resistant *C. albicans* isolates, but not the species shift to non-*albicans Candida* species in HIV patients.

A limitation of the study is that the lack of a 35°C incubator in the Chad laboratory might have influenced the *Candida* growth results, including the CFUs. In addition, the need to preselect colonies on site while performing species identification only after re-culture may have led to underestimation of non-*albicans Candida* species.

## Conclusion

The HIV-infected patients in Chad enrolled in our study presented with an oral yeast flora comparable to other sub-Saharan African countries with *C. albicans* being the predominant species. Oral candidiasis still remains a significant opportunistic infectious disease in advanced stages of AIDS with CD4^+^ T-cell counts <200 cells/μl. Under HAART, a significant reduction of the fungal burden in the oral cavity was seen. It is likely that HAART led to restoration of the immune system with a rise in CD4^+^ T-cell counts that protected the oral cavity against fungal colonization with *C. albicans*. Higher CD4^+^ T-cell counts also correlated with lower oral yeast colonization and the emergence of non-*albicans* species, possibly through repression of *C. albicans*. Antifungal resistance is not yet a concern in Chad. The intrinsically azole-resistant species *C. krusei* and *C. glabrata* were observed although a selection through azole treatment toward these species could not be demonstrated.

## Data Availability Statement

The original contributions presented in the study are included in the article/supplementary material; further inquiries can be directed to the corresponding author.

## Ethics Statement

The studies involving human participants were reviewed and approved by participating institutions in Chad and the ethical committee of the University Medical Center Göttingen, Germany (21/06/07). Written informed consent for participation was not required for this study in accordance with the national legislation and the institutional requirements.

## Author Contributions

OB and UG had the initial idea which was developed in a project together with TB, MS, and MW. LT-G, MK, WM, LK, MW, and OB collected the samples and performed the microbiological analyses. LT-G, WM, and LK interviewed the patients and together with all authors interpreted the results. All authors contributed to the article and approved the submitted version.

## Conflict of Interest

UG and OB have received financial support from Pfizer and Astellas Pharma.

The remaining authors declare that the research was conducted in the absence of any commercial or financial relationships that could be construed as a potential conflict of interest.

## Publisher’s Note

All claims expressed in this article are solely those of the authors and do not necessarily represent those of their affiliated organizations, or those of the publisher, the editors and the reviewers. Any product that may be evaluated in this article, or claim that may be made by its manufacturer, is not guaranteed or endorsed by the publisher.
